# Corrosion Behavior of Detonation Gun Sprayed Fe-Al Type Intermetallic Coating

**DOI:** 10.3390/ma8031108

**Published:** 2015-03-13

**Authors:** Cezary Senderowski, Michal Chodala, Zbigniew Bojar

**Affiliations:** Department of Advanced Materials and Technologies, Faculty of Advanced Technologies and Chemistry, Military University of Technology, 2 Kaliskiego St., Warsaw 00-908, Poland; E-Mails: mchodala@wat.edu.pl (M.C.); zbojar@wat.edu.pl (Z.B.)

**Keywords:** FeAl intermetallic coatings, D-gun spraying, corrosion resistance

## Abstract

The detonation gun sprayed Fe-Al type coatings as an alternative for austenitic valve steel, were investigated using two different methods of testing corrosion resistance. High temperature, 10-hour isothermal oxidation experiments at 550, 750, 950 and 1100 °C show differences in the oxidation behavior of Fe-Al type coatings under air atmosphere. The oxide layer ensures satisfying oxidation resistance, even at 950 and 1100 °C. Hematite, α-Al_2_O_3_ and metastable alumina phases were noticed on the coatings top surface, which preserves its initial thickness providing protection to the underlying substrate. In general, only negligible changes of the phase composition of the coatings were noticed with simultaneous strengthening controlled in the micro-hardness measurements, even after 10-hours of heating at 1100 °C. On the other hand, the electrochemical corrosion tests, which were carried out in 200 ppm Cl^−^ (NaCl) and pH ~4 (H_2_SO_4_) solution to simulate the acid-rain environment, reveal higher values of the breakdown potential for D-gun sprayed Fe-Al type coatings than the ones for the bulk Fe-Al type alloy and Cr21Mn9Ni4 austenitic valve steel. This enables these materials to be used in structural and multifunctional applications in aggressive environments, including acidic ones.

## 1. Introduction

Intermetallic compounds are interesting materials because of their unique properties, which are generally ascribed to their long-range ordered crystal structures [1,2,3,4,5,6].

Some of these materials possess high order near a critical temperature (*T*_c_) as high as their melting point, which allows them to maintain an ordered arrangement of the atoms and, thus, hinders diffusion processes at high temperatures [7].

Especially, iron aluminide-based intermetallic alloys are attractive materials for several industrial applications at medium to high temperatures [5,6,7,8,9], both as bulk materials and as coatings, because of their good mechanical properties, relatively low density (5.56 g/cm^3^ for the FeAl phase), excellent corrosion resistance in oxidizing and sulfidizing atmospheres (a result of their ability to form a highly protective Al_2_O_3_ scale [10,11]), and low manufacturing cost.

The bcc-based (body centered cubic) B2 ordered FeAl phase exists in the range of 36–50 at% Al and has a *T*_c_, which coincides with its melting point at 1250 °C. The properties of this compound include excellent oxidation, corrosion and sulfidation resistance, high electrical resistivity, reasonable strength from room temperature to about 500 °C and acceptable ductility at room temperature, partially dependent on environmental sensitivity [10].

Motivated by the above considerations, the present work addresses to the corrosion behavior of the iron aluminide FeAl–40 at% Al in the form of the D-gun spraying coatings under their thermal heating and electrochemical corrosion conditions.

According to the literature [8,9,10,11,12,13,14,15,16,17], FeAl type intermetallic protective coatings that are deposited by different thermal spraying techniques onto steel substrates possess very useful properties, like corrosion resistance at high-temperature, good mechanical properties, such as micro-hardness and adhesion, excellent lubricating abilities, tightness, low porosity, and excellent resistance to abrasive wear. Different elements of the power boilers in thermal-electric power plants have FeAl protective coatings that are deposited by high-velocity oxy fuel (HVOF) and D-gun spraying [10,11,12,13,18,19,20,21,22,23,24,25,26,27,28,29]. These coatings are designed to work in aggressive environments at elevated temperature and in abrasive wear conditions in fluidized-bed boilers [20,21,25].

Thermal spraying of iron aluminide coatings might also have applications in hot sections of gas turbine engines protection, however the coatings possess oxide inclusions and porosity, which can cause flaking of the coating. Especially, the high aluminum content in the Fe-Al type powders deposited on the structural steel will generate oxidation that usually occurs during thermal spraying, and may change the composition and microstructure design of the coatings, causing deterioration of the properties of the deposits [11,18,26,27,28].

In view of the above discussion, the objective of the present study is to determine results concerning the oxidation behavior of iron aluminide specimens, with coatings as-deposited self-decomposing FeAl–40 at% Al powders by D-gun spraying, except for the electrochemical corrosion resistance investigations.

## 2. Experimental

Commercially available self-decomposing FeAl feedstock powder with the composition Fe 58.5 at%, Al 40 at% and C 1.5 at% (spectral analysis), and the average particle size distribution between 38 and 75 μm was the starting material for D-gun spraying coatings. D-gun spraying process on the AISI (American Iron and Steel Institute) 1045 plain carbon steel was carried out at the optimum parameter values (discussed in [22]), which guarantee the repeatability of the metallic spray properties in each working cycle.

The AISI 1045 plain carbon steel, hardened and tempered, was grinded, stress relieved and finally, cleaned in abrasive blasting directly before coating [29].

The high temperature isothermal oxidation and thermal stability experiments of the FeAl type coatings was determined by heating in a furnace under air atmosphere for 10 h at 550, 750, 950 and 1100 °C, respectively.

The structural and physicochemical factors, such as the change in the morphology and chemical composition of the individual grains, phase change susceptibility and the degree of strengthening of the coating, were analyzed.

The analysis of inhomogeneity of the chemical composition (phase composition) of the FeAl coatings was carried out with Philips XL-30 scanning microscope (Philips Electron Optics, Amsterdam, The Netherlands) integrated with DX4i–EDAX X-ray microanalysis (Edax Inc., Mahwah, NJ, USA) as well as with Seifert XRD 3003 X-ray diffractometer (Seifert, Ahrenburg, Germany).

The porosity of the coatings was assessed by photomicrograph quantitative analysis carried out with (SEM) Philips XL30/Lab6 programmed with SIS software (Soft-Imaging Software GmbH, Münster, Germany). The Cavaleri-Hacquerta principle was applied [29], according to which the level of inner porosity of the D-gun sprayed coatings (*i.e*. cohesive porosity) is defined with planimetric method as the ratio of the sum of pore surfaces to the total surface of the specimen.

The assessment of the distribution of coating hardness in the layered structure was carried out according to the Vickers method with Shimadzu micro-hardness tester (Shimadzu, Kyoto, Japan) (load 100 g for 5 s).

**Figure 1:**
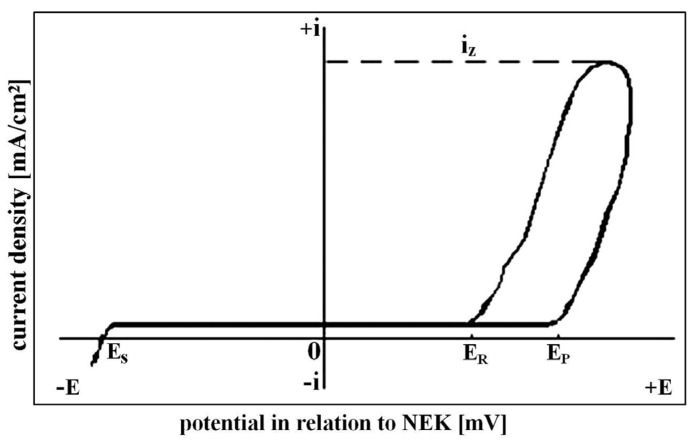
Scheme of the anodic cyclic polarization curves [29].

The corrosion resistance of the FeAl coatings after spraying was assessed by anodic cyclic polarization method in potentiodynamic and potentiostatic mode. The investigated sample is polarized by the potential, ranging from negative to positive values (anodic polarization) with simultaneous current flow record in the electrochemical cell. Anodic polarization curve (scheme in Figure 1) is in the current density (i)—applied potential (E) coordinates.

Before undergoing passivation with an appropriate electrolyte, the material begins at its stationary potential (*E*_S_); and next, it goes through the passivity range, where the material dilutes (material corrodes with a low, acceptable rate) at minimal, almost constant current density. When a certain value of the potential is overrun (*E*_p_—breakthrough potential) the passive layer is destructed and the investigated material undergoes pitting corrosion.

When a certain value of current density is reached, exhibiting intensive pits propagation, the direction of polarization is reversed to the cathode to describe ability of the material to recreate the passivity.

The quantity describing that ability is the re-passivation potential *E*_r_ (when current density reaches again the level of the passive state). The difference *E*_p_ − *E*_r_ is treated as a parameter describing resistance of a given material towards crevice corrosion.

In general, for the best local corrosion resistance, the differences *E*_p_ − *E*_s_ and *E*_r_ − *E*_s_ should be positive and as high as possible. The *E*_r_ value should not be lower than *E*_s_, *E*_p_ − *E*_r_, the difference should be minimal, and E_p_ value as high as it is possible.

The electrochemical corrosion resistance test is a typical comparative method and gained results are reliable when the same tests conditions are preserved.

Prior to the anodic polarizations, the open cell potential (*E*_S_) was being recorded for 6 h to stabilize the starting point for further measurements.

Recording the anodic polarizations curves were accomplished by using the UPE-3 set connected to the A/C-PCL-711CS converter at a constant potential change rate equal to 1 mV/s. The experiments were started 300 mV below the stationary potential *E*_s_. While the breakthrough potential was exceeded, the direction of the potential change was reversed as the assumed value of current density was gained. For every coating, three corrosion test curves were recorded. The third ones were taken under consideration.

An acidic solution of chlorides was applied as an electrolyte serving as a corrosive environment. The electrolyte contained 200 ppm Cl^−^ (NaCl) with sulfuric acid (H_2_SO_4_) at a pH of 4. It was a simulation of the atmospheric falls—acid rain.

Platinum grid was serving as a counter electrode with the investigated material inside. The saturated calomel electrode (SCE) was the reference electrode.

The experiments were conducted at room temperature. The samples were flat with uniform working surface area. All samples were polished with polishing paper (600 granulation), and sealed in epoxide resin with connected electric contact. During the experiments, electrolyte was stirred and in contact with air.

## 3. Results and Discussion

### 3.1. As-Detonation Gun Sprayed FeAl Coatings Characterization

**Figure 2:**
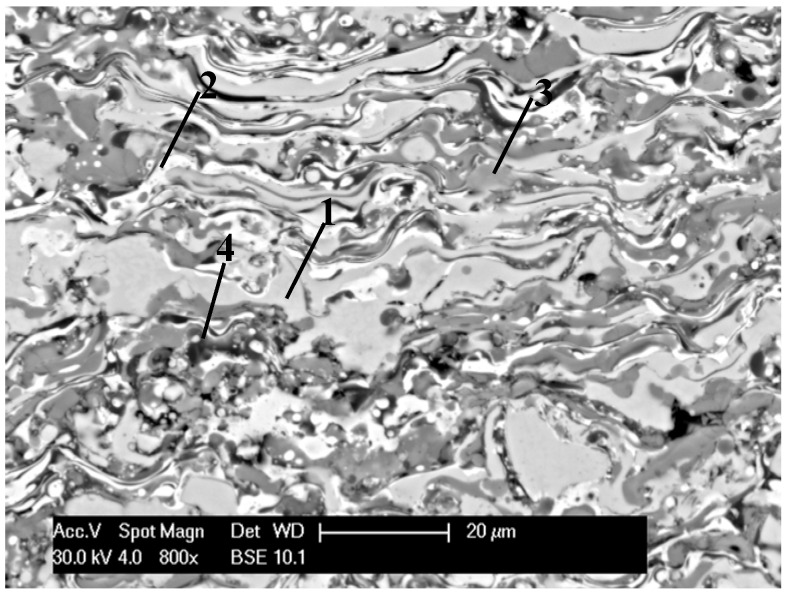
Morphology on the cross section of strongly deformed and oxidized, dispersive grains of the D-gun FeAl type coating, deposited in “high” value of the detonation energy.

The detonation mixture energy directly influences the metallurgical quality and geometrical parameters of the coating. The research proved that even insignificant changes in one of the parameters of D-gun spraying (such as the volume of fuel, oxidant and carrier gases, the spraying distance or frequency) considerably influenced the value of kinetic and thermal energies of the process, which are decisive factors for the quality of D-gun sprayed coatings. It was found that the change of the process parameters (pressure: propane-butane 0.01–0.028 MPa, oxygen 0.004–0.017 MPa, nitrogen 0.001–0.006 MPa, spraying distance 160–250 mm and the frequency of spraying 3–6 Hz), result in considerably different values of detonation energy, classified as “high”, “typical” and “low” [22]. When the sprayed powder particles had high energy, the temperature of the particles was high and obtained coatings had highly deformed and oxidized grains (Figure 2). Conducted microanalysis of the chemical composition in the coatings micro-areas confirmed the presence of the alumina thin films (Al_2_O_3_), especially in the surface area of the coatings, see Table 1. Also highly oxidized particles (various oxidation level) were present in the coatings volume.

Presented results show that at high spraying energy, major oxidation of the FeAl surface grains takes place. It undoubtedly improves corrosion resistance. However the phenomenon can be disadvantageous for metallic coatings, because presence of oxides in the intermediate area in the support-coating boundary can cause adhesion to decrease, crucial for the FeAl brittle coatings application. Moreover, strong oxidation of the coating grains can also cause total dissection of the coating, resulting in decohesion.

**Table 1:** Microanalysis of the chemical composition of D-gun sprayed Fe-Al type coating deposited in high spraying energy (grains area according Figure 2).

Grain area	Remarks	Content (at%)		
		Fe	Al	O
Light grey (1)	FeAl lightly oxidized	43.5	50.1	6.4
White (2)	FeAl/Fe_3_Al lightly oxidized	54.2	38.1	7.7
Grey (3)	FeAl strongly oxidized	36.7	57.4	16.9
Dark grey (4)	Al_2_O_3_ oxide films in FeAl coating	31.9	32.7	35.4

**Figure 3:**
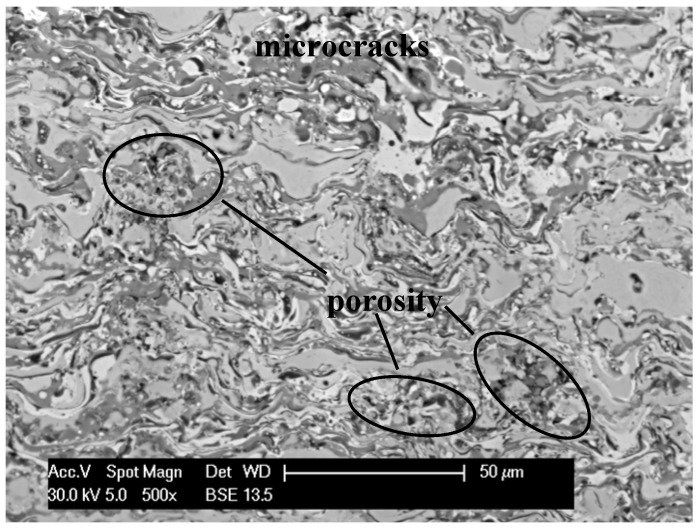
Microstructure on the cross section of the D-gun sprayed Fe-Al type coating deposited in “low” value of the detonation energy (characteristic porosity and microcracks near top of the coating).

It was found that the application of powder carrier gas (nitrogen 2%–5%) reduces the effect of oxygen activity in the metallic spray (less oxides in the coating volume). It also reduces the temperature and exit velocity of detonation products, which in turn considerably influence kinetic energy of powder particles [22]. This energy is a decisive factor, which determines the quality of the structure of the FeAl coatings and particularly their porosity (Figure 3). The value of cohesive porosity was 2%–5% with a tendency to grow along with the volume increase of the carrier gas (nitrogen).

Eventually, it turned out that depositing a coating that exhibits lamellar structure, low porosity (below 0.5%), and repeatable thickness was relatively easy with the following parameters: propane-butane 0.017 MPa, oxygen 0.009 MPa, nitrogen 0.003 MPa, spraying distance of 240 mm, and frequency of 5 Hz.

**Figure 4:**
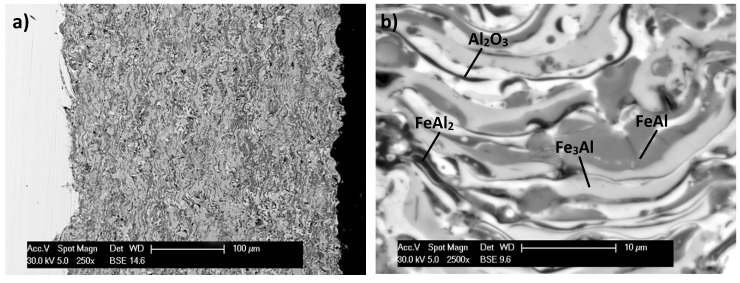
Surface layer of FeAl/1045 steel exhibiting thickness microstructure (**a**) and grain morphology (**b**)—on the cross section of the D-gun sprayed Fe-Al type coating deposited in “typical” value of the detonation energy.

The coatings deposited are built of lamellar splats formed by the powder particles, which undergo strong plastic deformation and geometrical changes during their transformation into the coating material (Figure 4).

As a result of the analysis of morphology and phase allocation of D-gun sprayed FeAl type coating, it was found that the coating have a microstructure typical for the D-gun spraying method [11,13,26]. Their structure consists of layered and flattened grains of the intermetallic phases from the Fe-Al diagram with predominant FeAl phase. The coatings, however, have varied chemical composition (according to the diagram the compositional range of the FeAl intermetallic is wide and ranges from 36 to 50 at% Al).

On the basis of the point EDS (Energy Dispersive X-ray Spectroscopy) analysis, the low-aluminum Fe_3_Al phases (the brightest areas in Figure 4) and the FeAl_2_ phases (dark grey in Figure 4) were identified where the aluminum content level exceeds the upper range of FeAl phase. It should be emphasized that the “composite” nature of D-gun sprayed Fe-Al type coatings is triggered by the existence of dispersive intermetallic phases with different ordering (depending on the aluminum contents) and especially considerable participation of oxygen influencing the creation of stable and durable oxide layers (mainly Al_2_O_3_).

**Figure 5:**
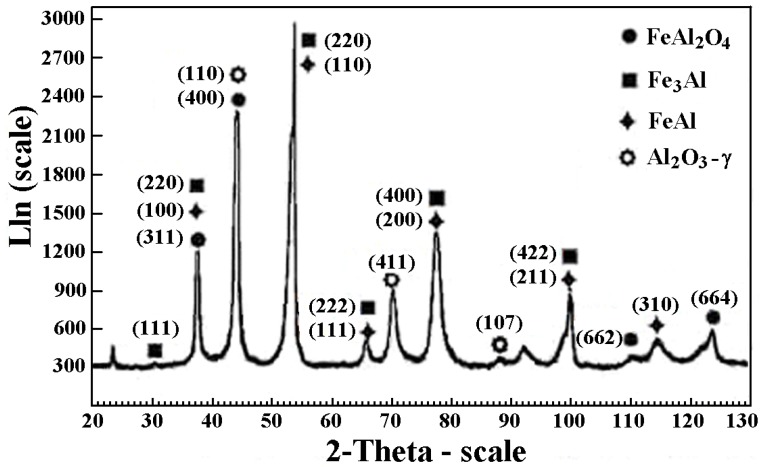
X-ray diffraction (XRD) pattern of detonation Fe-Al type coating sprayed on AISI 1045 carbon steel.

The X-ray phase analysis of the FeAl D-gun spraying coatings (Figure 5) confirmed that the intermetallic FeAl phase as the basic component of the structure is “inherited” from the powder. The results confirmed also that the coating contains an oxide Al_2_O_3_ and a complex oxide Fe + 2Al_2_O_4_, the fraction of which is comparable to that of the metallic FeAl matrix, which was identified after heating at 950 or 1100 °C as approximately equivalent to Al_2_O_3_ [26]. The aluminum-rich Fe-Al powder is so chemically active in the D-gun spraying process that all powder particles are already oxidized and the deposited coatings always contain oxide films inside the coating and at the internal interfaces [11,26]. It is the formation of the oxide films identified on the basis of the point EDS analysis (the darkest areas of the coating structure (Figure 2 and Figure 4)), which brings about the lamellar structure of the FeAl intermetallic coatings and defines their composite character [26].

**Figure 6:**
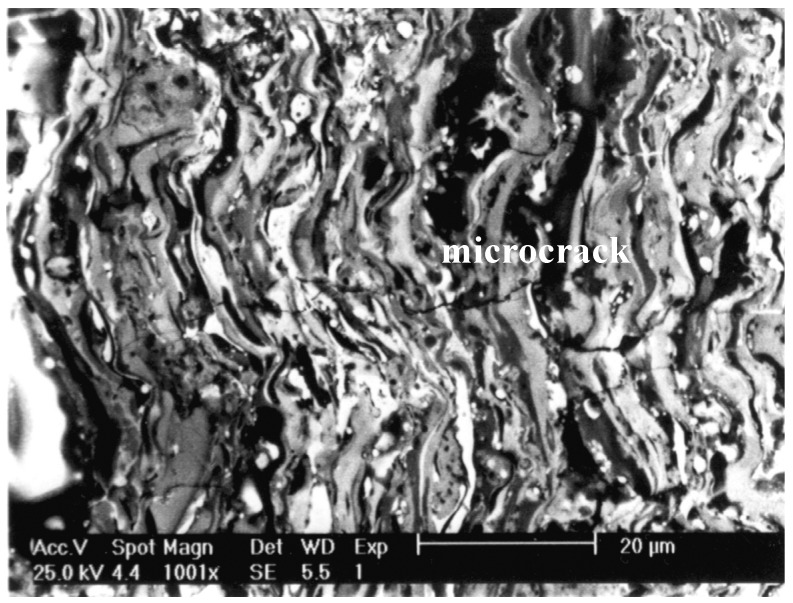
Microstructure on the cross section of the D-gun sprayed Fe-Al type coating deposited in “high” value of the detonation energy (characteristic microcracks in volume of the coating) [29].

Most probably it is the presence of Al_2_O_3_ scales and high ordering of intermetallic phases with increased amount of aluminum coupled with high kinetic energy of D-gun spraying that is the reason of the formation of visible microcracks (Figure 6) running perpendicularly to the lamellar splats [22,29]. This, in turn, proves that the splat boundaries are not prone to development of cracks and have high degree of cohesive strength. One can also assume that the crack resistance of the identified earlier FeAl phases with different oxidation degree is similar, as the microcracks run through several layers of the coating splats (their usual length is up to 25 μm). Perpendicular orientation of the microcracks to the coating surface allows one to infer that the cracking is caused by dominating thermal tensile stresses in the condition of high brittleness of the phases and is initiated during coating cooling down, as a result of difference in linear coefficients of thermal expansion which equal α = (18 × 10^−6^) for FeAl and α = (12 × 10^−6^) for steel [22].

### 3.2. Electrochemical Corrosion Behavior

**Figure 7:**
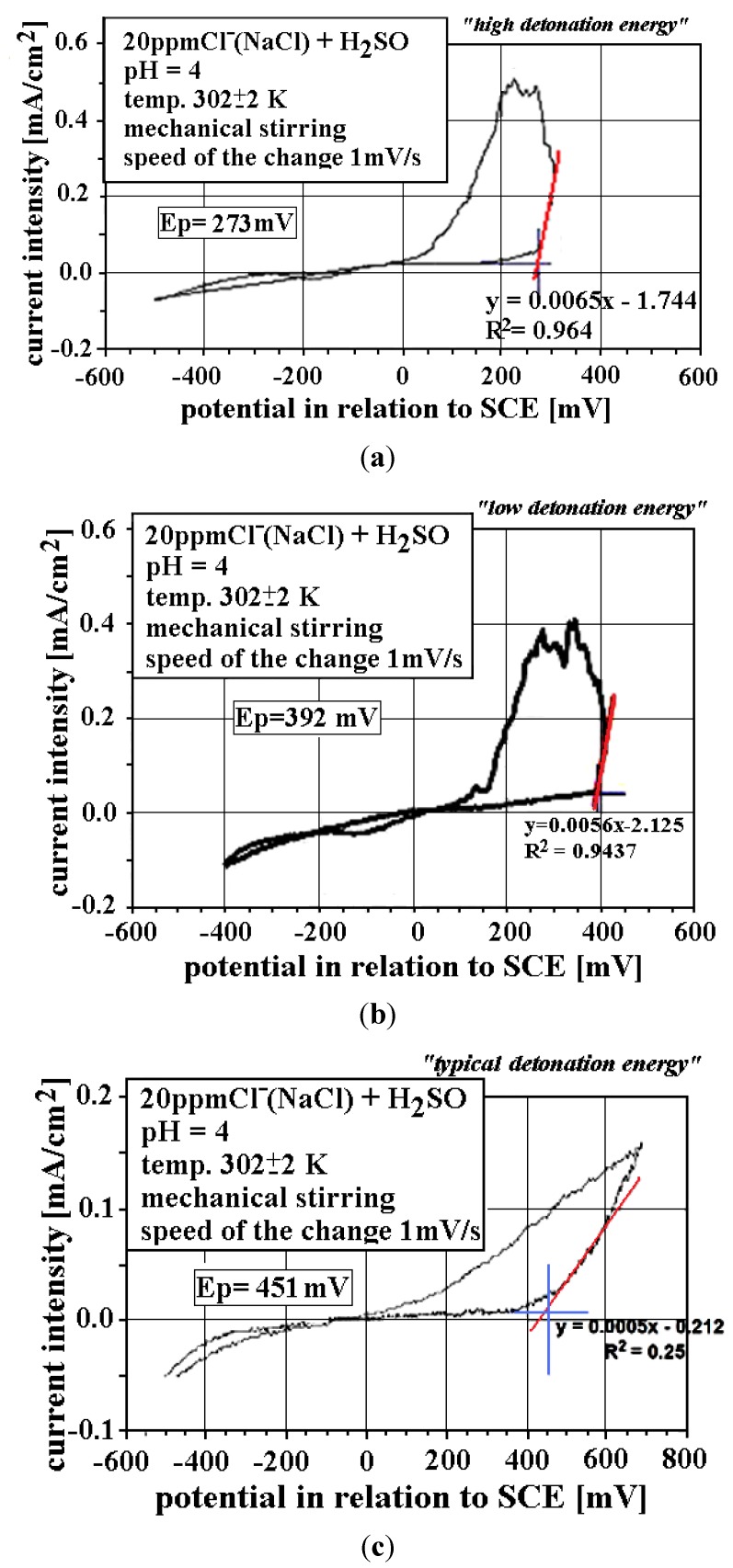
Anodic cyclic polarization curves for FeAl D-gun spraying coatings at the following experimental conditions: 200 ppm NaCl + H_2_SO_4_, pH = 10, *t* = 302 ± 2 K, mechanical stirring, potential change rate—1 mV/s (**a**); (**b**) FeAl coatings with higher porosity and individual microcracks; and (**c**) FeAl coating with porosity below 0.5% and higher part of Al_2_O_3_ oxides.

**Figure 8:**
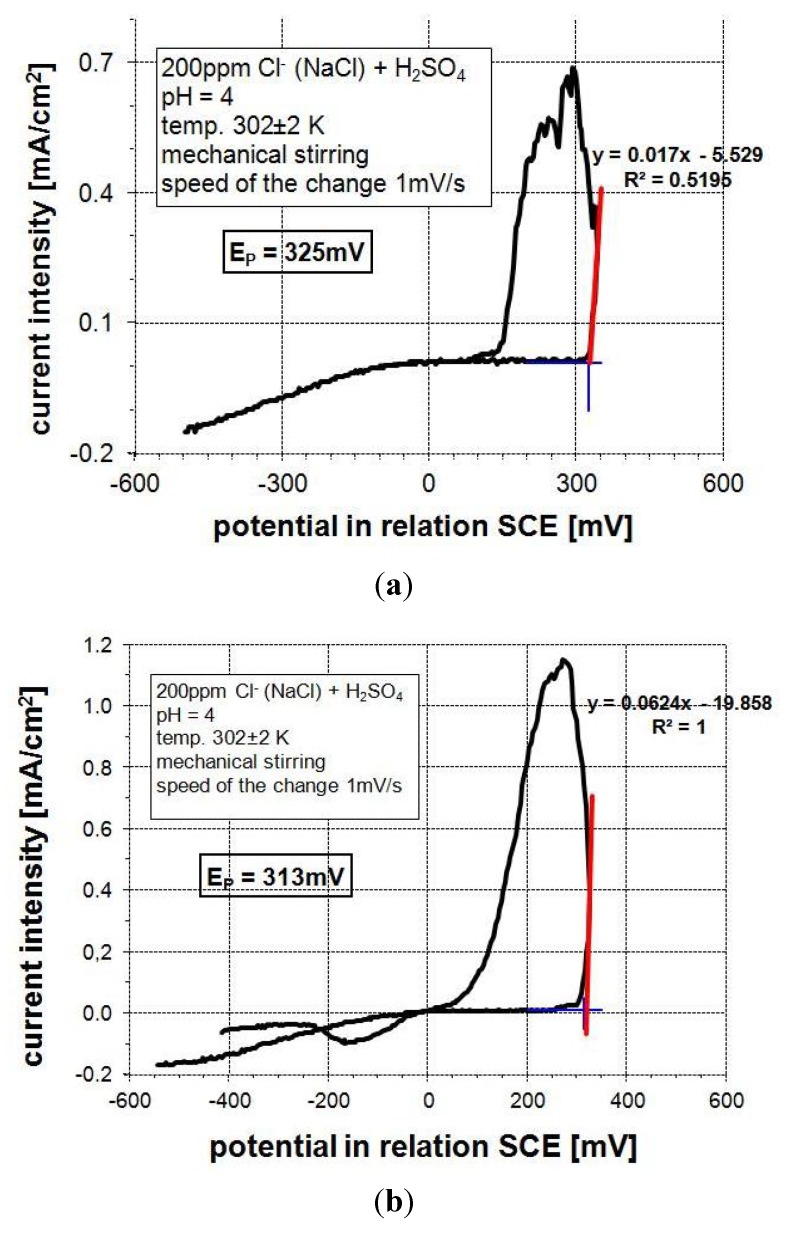
Anodic cyclic polarization curves for bulk materials at the following experimental conditions: 200 ppm NaCl + H_2_SO_4_, pH = 10, *t* = 302 ± 2 K, mechanical stirring, potential change rate—1 mV/s; (**a**) alloying cast with Fe_3_Al matrix with chrome, nickel, vanadium, boron and niobium as additions; and (**b**) valve steel 50H21G9N4.

Corrosion resistance experiments of the FeAl coatings (Figure 7) were performed by using cyclic anodic polarization in the acidic electrolyte containing 200 ppm chlorides—Cl^−^ (NaCl) + H_2_SO_4_ (modeling of the acid rain environment). According to the literature, the surface characteristics of thermal sprayed coatings are very relevant to the value of the electrochemical corrosion. Those observations have been made by Guilemany *et al.* [30] during examinations of corrosion behavior of the HVOF and APS (Atmospheric Plasma Spraying) sprayed nitinol coatings. They have shown that in the as-polished HVOF coatings, the *E*_corr_ potential values decrease considerably, compared to the same coatings in the as-sprayed form (without polishing). The results have been attributed to the formation of cracks after polishing or oxide removal on the top layer of HVOF coatings. According to this, the corrosion resistance experiments have been performed on the polished surface of the FeAl coatings with the surface roughness (*R*_a_) below 2 μm. The tests revealed that obtained D-gun sprayed coatings have satisfying resistance towards pitting corrosion in comparison to the bulk samples made of Fe_3_Al intermetallic alloy with additions of chrome, nickel, vanadium, boron and niobium (Figure 8a) and to the valve steel 50H21G9N4, material applied in the combustion engines construction (Figure 8b).

Analysis of the anodic polarization curves (Figure 7a,b) shows that the lowest breakthrough potential (respectively, *E*_p_ = 273 and 392 mV in relation to SCE (Saturated Calomel Electrode) was for FeAl coating where high chemical and phase inhomogenity and the highest porosity were found. The lowest breakthrough potential confirms the ease of pitting corrosion initialization.

The relatively low level of corrosion resistance of the FeAl coating with low quality metallurgical features can be attributed to its penetrability, including micro-cracks providing easy penetration of the sample by the electrolyte (Figure 6). The highest values of the breakthrough potential (*E*_p_ = 451 mV for SCE (Figure 7c)) are for FeAl coating where the highest content of aluminum and the lowest porosity (below 0.5%) were found. The presence of alumina thin films serving as a protector for FeAl, the major component of the coatings matrix was noticed.

Generally, the breakthrough potential values (*E*_p_) of the investigated D-gun sprayed coatings (excluding mechanically penetrable samples with low metallurgical features) are higher from ones for Fe-Al bulk material (Figure 8a) and high-alloying valve steel 50H21G9N4 (Figure 8b). The coatings can be dedicated to the structural applications in the acidic environments and aggressive corrosion environments.

### 3.3. Corrosion Performance in High Temperature Air Environments

The next stage of the research was the evaluation of the corrosion resistance and thermal stability of the FeAl coating after D-gun spraying and additional heating for 10 h at 550, 750, 950 and 1100 °C, respectively.

**Figure 9:**
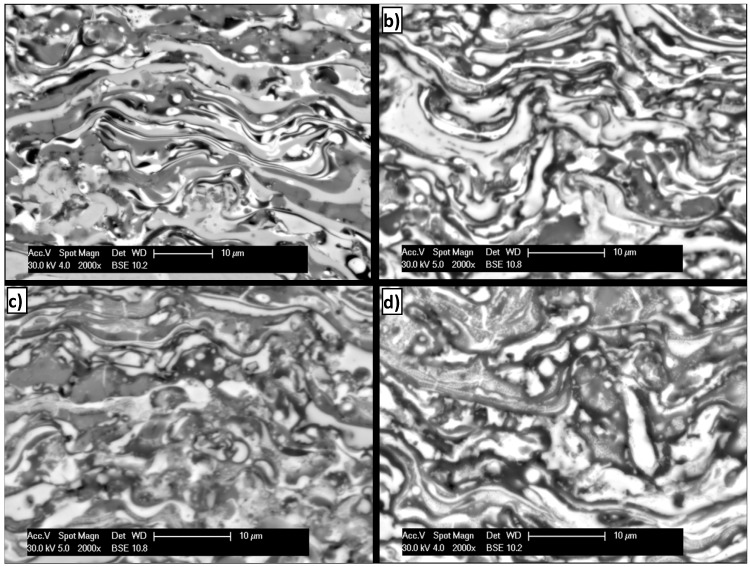
Typical microstructure on the cross section of detonation sprayed FeAl intermetallic coating and after additional heat treatment at (**a**) 550 °C; (**b**) 750 °C; (**c**) 950 °C; and (**d**) 1100 °C for 10 h.

The macroscopic view on the coatings shows no damages after 10-hour heating at 550 °C and 750 °C (Figure 9a,b). Ten-hour heating at 950 and 1100 °C enlarges areas where splats fragmentation occurs (Figure 9c,d); simultaneously layered structure type of the coating is conserved. After heating at 950 and 1100 °C, fragmentation of the coatings structure into axial grains (<1 μm) was noticed, especially in the area of splats with composition of secondary FeAl solution with diverse oxidation numbers (Figure 9c,d).

**Figure 10:**
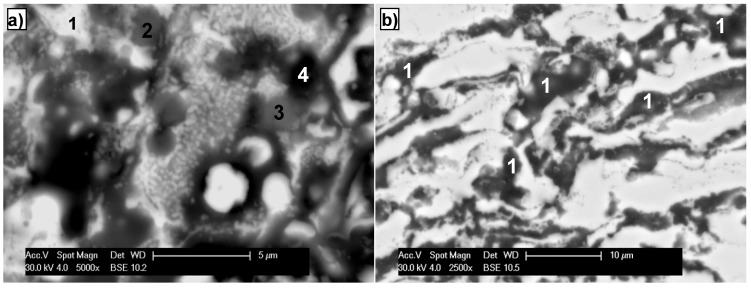
Example microstructure on the cross section of detonation sprayed FeAl intermetallic coating and after additional heat treatment at 750 °C for 10 h; hypothetical phase identification based on results of (**a**) EDS point analysis; and (**b**) dispersive Al_2_O_3_ oxides.

Diverse phase composition of the splat structure of the coating after high temperature heating was evaluated basing on the BSE images. Four basic types of layered grains were analyzed, marked as (1), (2), (3) and (4)—Figure 10a.

In general, high temperature heating implies homogeneity of the chemical composition of the FeAl coatings grains. The brightest phase (1) has the highest content of iron. In the areas of “bright gray”, (BSE image) phase (3) aluminum is the dominating element. In areas (2) and (4), which are “dark gray” and “dark”, respectively, oxygen is the dominating element (Figure 10a). In some grains of the FeAl coating, dispersed particles of oxides, mainly alumina, are present locally (Figure 10b point 1).

**Figure 11:**
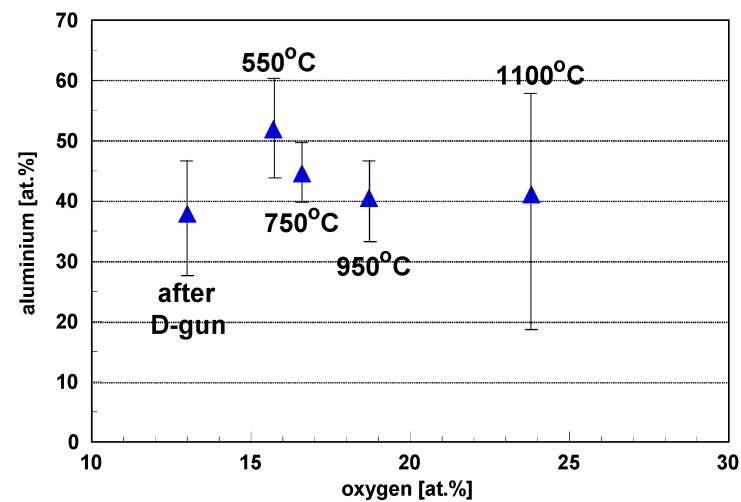
Average values and standard deviations of aluminum and oxygen content based on results of EDS point analysis of FeAl intermetallic coating after D-gun spraying and additional heating treatment, respectively, at 550, 750, 950 and 1100 °C for 10 h.

Figure 11 shows chemical homogeneity of the FeAl coating after high temperature heating confirmed by statistical analysis, where average values were estimated from point analyses. Systemization of the EDX results for FeAl coatings heated in four heating temperatures: 550, 750, 950, 1100 °C enabled showing the tendency in the area of co-dependency aluminum and oxygen participation in the FeAl coating as a function of the heating temperature. Results reveal that heating causes relative increases of aluminum and oxygen content in comparison to the coating-state directly after spraying. This is the result of much easier passivation of the high-aluminum phases at elevated temperatures and connected thickening alumina thin films.

Moreover, statistical analysis of gained results exhibited (especially for standard deviation values) smaller dispersion of the aluminum content, especially after heating at 750 °C, which confirms chemical homogeneity increase in the layer of FeAl grains, mainly covered by complex alumina thin films, resulting from high temperature heating.

**Figure 12:**
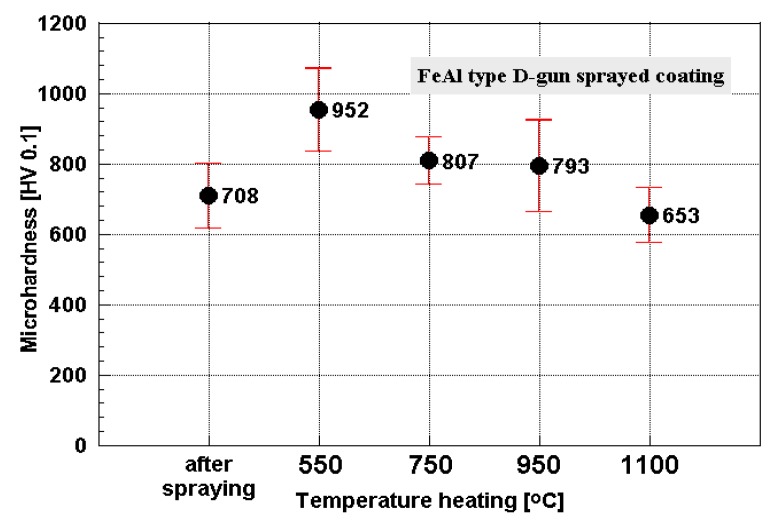
Average microhardness of FeAl type coating *versus* heat treatment temperature [29].

Ten-hour heating (550, 750, 950 °C) causes the effect of gradual homogeneity increase of the chemical composition and structure, and results in a major increase in microhardness of the FeAl coatings (Figure 12). The average coating material microhardness was exceeded by 650 HV 0.1, also after heating at 1100 °C, despite decrease of the coating average micro-hardness (Figure 12). Supersaturation of the heated FeAl coatings with vacancies at 550 and 750 °C results with noticeable increase of the microhardness (even during slow cooling in the turned-off oven).

Results of the strengthening level confirm high thermal stability of the FeAl coatings.

**Figure 13:**
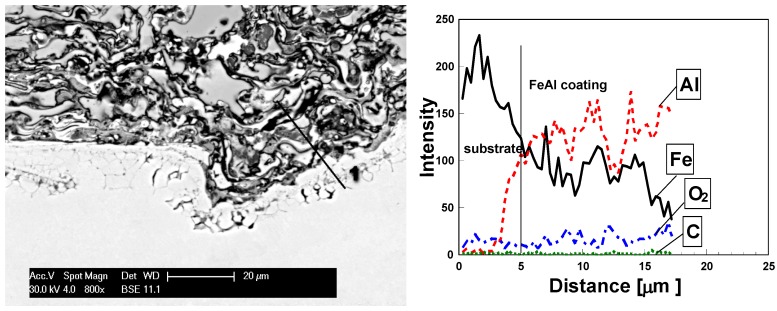
Linear distribution of elements in the FeAl coating after heat treatment at 1100 °C for 10 h.

A slow diffusion process of aluminum towards support surface layer (steel 1045) was noticed and confirmed by linear analyses (Figure 13) of the chemical composition for coating/support boundary of the coating heated at 1100 °C.

An intermediate layer with high adhesion, simultaneously preventing aluminum diffusion toward supporting material should be potentially applied [26]. Small diffusion inside the FeAl coating can be advantageous because of homogeneity and increases cohesion resistance of the grain boundaries, serving as a kind of thermal barrier.

## 4. Conclusions

(1) Limited chemical interactions between detonation gas products and coating material can not be avoided in the D-gun spraying conditions.
(2) High stability of the intermetallic powders particles allows conserving phase composition, but high content of aluminum particles are being covered by thin oxide films (mainly alumina) during flight to the treated surface.
(3) At the minimal thickness, oxide film and layer coating structure, presence of the oxides does not affect the mechanical properties of the material, but is advantageous in the aspect of corrosion resistance.
(4) Electrochemical corrosion resistance of the sprayed FeAl coatings at optimal spraying conditions is higher for low porosity coating than for bulk Fe-Al and austenitic valve steel Cr21Mn9Ni4. It allows concluding that the coatings may find applications in aggressive environments containing acids.
(5) An important attribute of the fabricated coatings is their corrosion resistance and thermal stability in high temperatures. After heating at temperatures range from 550 to 1100 °C for 10 hours, negligible changes in phase composition and coating morphology were found. Strengthening, controlled by micro-hardness measurements, was fully conserved.
